# Societal discrimination and mental health among transgender athletes: a systematic review and Meta-analysis

**DOI:** 10.1186/s40359-023-01493-9

**Published:** 2024-01-16

**Authors:** Alex Siu Wing Chan, Alston Choong, Kean Chang Phang, Lok Man Leung, Patrick Ming Kuen Tang, Elsie Yan

**Affiliations:** 1 Department of Applied Social Sciences, Faculty of Health and Social Sciences, The Hong Kong Polytechnic University, Hong Kong, SAR China; 2 Department of Sports Medicine, Faculty of Medicine, University of Malaya, Kuala Lumpur, Malaysia; 3 Department of Pathology, Faculty of Medicine, University of Malaya, Kuala Lumpur, Malaysia; 4 Department of Anatomical and Cellular Pathology, State Key Laboratory of Translational Oncology, The Chinese University of Hong Kong, Hong Kong, SAR China

**Keywords:** Transgender, Inequality, Mental health, Sport, Minorities stress, Health promotion, Health care

## Abstract

**Background:**

Discrimination and inequality have been identified as significant problems faced by transgender individuals in sports participation. However, uncertainties remain regarding the effectiveness of interventions aimed at promoting equality.

**Objectives:**

This systematic review and meta-analysis aimed to examine the experiences of transgender athletes in sports, focusing on mental health issues and factors contributing to inequality among transgender and other sexual minorities.

**Methods:**

The study followed the Preferred Reporting Items for Systematic Reviews and Meta-Analyses (PRISMA) guidelines and searched 10 electronic databases, including PubMed, Google Scholar, and Web of Science, to identify eligible studies published between 2005 and 2022. The search yielded 1430 articles, of which only 12 studies met the inclusion criteria for this review.

**Results:**

The meta-analysis of the 12 studies included in this review revealed that transgender athletes faced social discrimination and inequality in sports participation, resulting in mental health problems and higher rates of suicide. From a cohort of 21,565 participants in the studies, 7152 (33%) were subjected to discrimination in sports participation and healthcare, with a rate of 0.61 (95% confidence interval [CI]: 0.35, 0.81). However, transgender athletes who felt welcomed and embraced by their respective teams accounted for 0.39 (95% CI: 0.19, 0.65). These results indicated significant differences between how transgender athletes are treated in healthcare settings and when participating in sports.

**Conclusion:**

The study findings underscore the need for policies, cultural research, and interventions to address discrimination and inequality faced by transgender athletes in sports participation. Promoting equality and safeguarding the rights of transgender athletes can mitigate the risk of mental health problems and increase physical activity among sexual minorities.

## Background

Sexual minorities, including lesbian, gay, bisexual, and transgender (LGBT) individuals, often encounter difficulties when participating in sports, as evidenced by comprehensive research [1–3]. These disparities include inadequate healthcare, exclusion from certain activities, discrimination, mental health issues, and frustration. Transgender individuals face daily psychological, practical, political, and physical challenges that are widely recognized and experienced at all levels [1]. As such, there is a growing need for research to explore the experiences of transgender individuals in sports and how they are treated as two-sex individuals on different occasions [4, 5]. Much research has been conducted on the problems faced by transgender individuals when participating in sports, including evaluations and regulations concerning the participation of sexual minorities. However, some studies have reported significant progress in the inclusion of transgender people in sporting activities [6]. These studies have played a substantial role in bringing to light and addressing the difficulties faced by transgender people, especially considering that participation in sports is an essential aspect of their daily lives [7]. Discrimination of sexual minorities in society can be linked to social inclusion in sports arenas, which has been a major topic of policy involvement and academic concern [8–10]. Additionally, the role of sports in facilitating or inhibiting the social participation of individuals with different sexual orientations, such as transgender people, has not been adequately addressed in policy-making [11, 12].

Reviewing sport policies for inclusiveness towards transgender competitors involved a systematic assessment of their fairness, considering available physiological research on athletic advantage [13]. In 2004, the International Olympic Committee (IOC) introduced a policy allowing transgender individuals who transition after puberty to compete in line with their experienced gender, provided they undergo gender-confirming surgery, show legal gender recognition, receive cross-sex hormone treatment for at least 2 years, and live in their experienced gender for the same duration. Transgender individuals undergoing surgery pre-puberty are also eligible under this policy, which is globally adopted by sports organizations [13, 14].

While praised for inclusivity, the 2004 IOC policy has identified shortcomings. It excludes transgender individuals opting out of surgery due to reasons like a lack of genital distress, medical concerns, or personal choices. Additionally, individuals in the transitioning process, receiving cross-sex hormones but yet to undergo surgery, are excluded [13, 14]. The policy’s narrow focus on transgender females raises concerns of discrimination against transgender males. Furthermore, it overlooks regional and international variations in accessing hormone treatment and surgery, lacking an evidence-based rationale for the 2-year hormone treatment period. The 2-year duration may be linked to historical IOC doping sanctions. The rationale for gender-confirming surgery is unclear, as genital anatomy doesn’t affect physiological advantages. The role of testosterone blockers in transgender women is also unaddressed.

The lack of participation of transgender youths in sports and physical activities may be attributed to traditional gender beliefs associated with sports and athleticism, which can impact their self-esteem [15, 16]. However, young sexual minorities frequently engage in homophobia to prove their heterosexuality and masculinity, enforcing social gender norms. Sexual judgment is pervasive when it comes to athletic participation, making sporting environments unsafe and unwelcoming for most sexual minorities [15, 17]. For healthcare workers at patient care centers, patient health and well-being are the main driving forces for all care system decisions. Therefore, patients should be treated from a mental, emotional, financial, societal, and spiritual perspective, rather than treating them solely based on a physiological perspective [17]. The negative impacts of heteronormativity, which lead to the perception of heterosexuality as the default or norm of sexuality, have been documented in various medical institutions [18, 19]. Therefore, assuming that all individuals are cisgender promotes a negative mentality toward sexual minorities in healthcare institutions. It is essential to eliminate cisnormativity beliefs and mentalities in medical and healthcare institutions related to sexual minorities, such as transgender patients, to promote equality among all patients [20–22].

Recent data indicates a substantial rise in the utilization of transgender health services by transgender individuals across various European countries [23, 24]. Moreover, there has been a noteworthy increase in the number of people self-identifying as transgender, irrespective of their attendance at transgender health services [25]. For instance, Kuyper and Wijsen [26] uncovered that 4.6% of individuals assigned male at birth and 3.2% of those assigned female at birth in a Dutch population sample reported having an ambivalent gender identity, where they identified equally with the gender they were assigned at birth and the other gender. The same study also found that 1.1% of individuals assigned male at birth and 0.8% of those assigned female at birth identified as transgender. It remains uncertain how many of these individuals will eventually seek treatment through transgender health services. This upsurge in people identifying as transgender may, in part, be attributed to the growing visibility of transgender individuals in Western society [27]. Notably, the public announcement by Caitlin Jenner, a former athlete and current television personality, about her transgender identity during a widely viewed television interview, has contributed to increased visibility [28]. Such heightened visibility may have prompted individuals to reflect on and question their own gender identity [6, 29].

Several initiatives and campaigns have been conducted to put an end to disparities in healthcare and mental health treatment among sexual minorities. However, the organizations spearheading these campaigns, such as the U.S Department of Health, are yet to achieve this goal. Various organizations, such as the National Collegiate Athletic Association (NCAA), have implemented rules and regulations to safeguard transgender athletes and protect their rights to participate in sports [30, 31]. However, there have been no published reports on whether healthcare providers or transgender individuals are aware of their rights to these protections. Such limitations have resulted in transgender people and other sexual minorities being subjected to unfriendly environments and feeling unwelcome to participate in sporting activities, leading to a detrimental or negative impact on their mental and physical health [31]. Fortunately, because of frequent interactions with transgender patients, athletic trainers (ATs) are trying to bridge the gap associated with sexual minorities in healthcare, with efforts to end inequalities among transgender people. Such measures can be achieved by promoting equality, making them feel comfortable, and creating a discrimination-free society that is safe for their mental health [32–34]. Consequently, this study aimed to explore the experiences of transgender athletes in sports, along with various themes to evaluate the mental health issues and factors that promote inequalities among transgender people and other sexual minorities. By understanding these experiences and factors, we can work towards promoting a more inclusive and welcoming environment for all individuals in sports and healthcare. It is crucial to acknowledge the unique challenges faced by sexual minorities and to work towards creating a society that is free from discrimination and inequality. Through ongoing research and advocacy, we can create a world where all individuals, regardless of their sexual orientation or gender identity, can participate in sports and receive quality healthcare without fear of discrimination or exclusion.

## Methods

### Study design

The inequality and mental health status of transgender athletes were assessed, identified, and reviewed using a multi-process strategy. Studies exploring pertinent information were carefully searched to examine the mental health and inequalities of transgender individuals in sports arenas. Studies detailing the inequalities among transgender sports people were grouped and analyzed to identify and establish the disparities in sports and mental health issues among transgender people and other sexual minorities.

### Information sources and searches

This systematic review and meta-analysis was conducted following the protocols stipulated by the (PRISMA) guidelines [34]. An initial information search was conducted using ten electronic databases: PubMed, Google Scholar, Web of Science, Medline, Cochrane, Springer, PsycINFO, Scopus, JSTOP, and IEEE Xplore. When searching for relevant information and primary studies, particular keywords and expressions relevant to the topic were used. The search criteria used the following logical conditions: (inequality OR mental health) AND (transgender athletes. The reference list was scored for additional pertinent studies. The keywords used in the search were “Transgender,” “Sports Participation,” “Mental Health,” and “Inequalities.” The same set of keywords were used for all the databases to ensure consistency in the search.

### Eligibility criteria

Three reviewers (A.S.W.C., P.M.K.T., and L.M.L.) determined the relevance of the included studies by assessing them using specific eligibility criteria. The reviewers applied the exclusion and inclusion criteria to screen the studies retrieved from the ten electronic databases. Studies included in the review were published in the English language between 2005 and 2022, and focused on transgender individuals in sports and athletic activities. Studies published in languages other than English, before 2005, or those that had transgender participants in fields other than sports were excluded. The primary outcome of interest was inequality and mental health among transgender individuals, and studies that focused on other sexual minorities were excluded.

### Search strategy

**Fig. 1:**
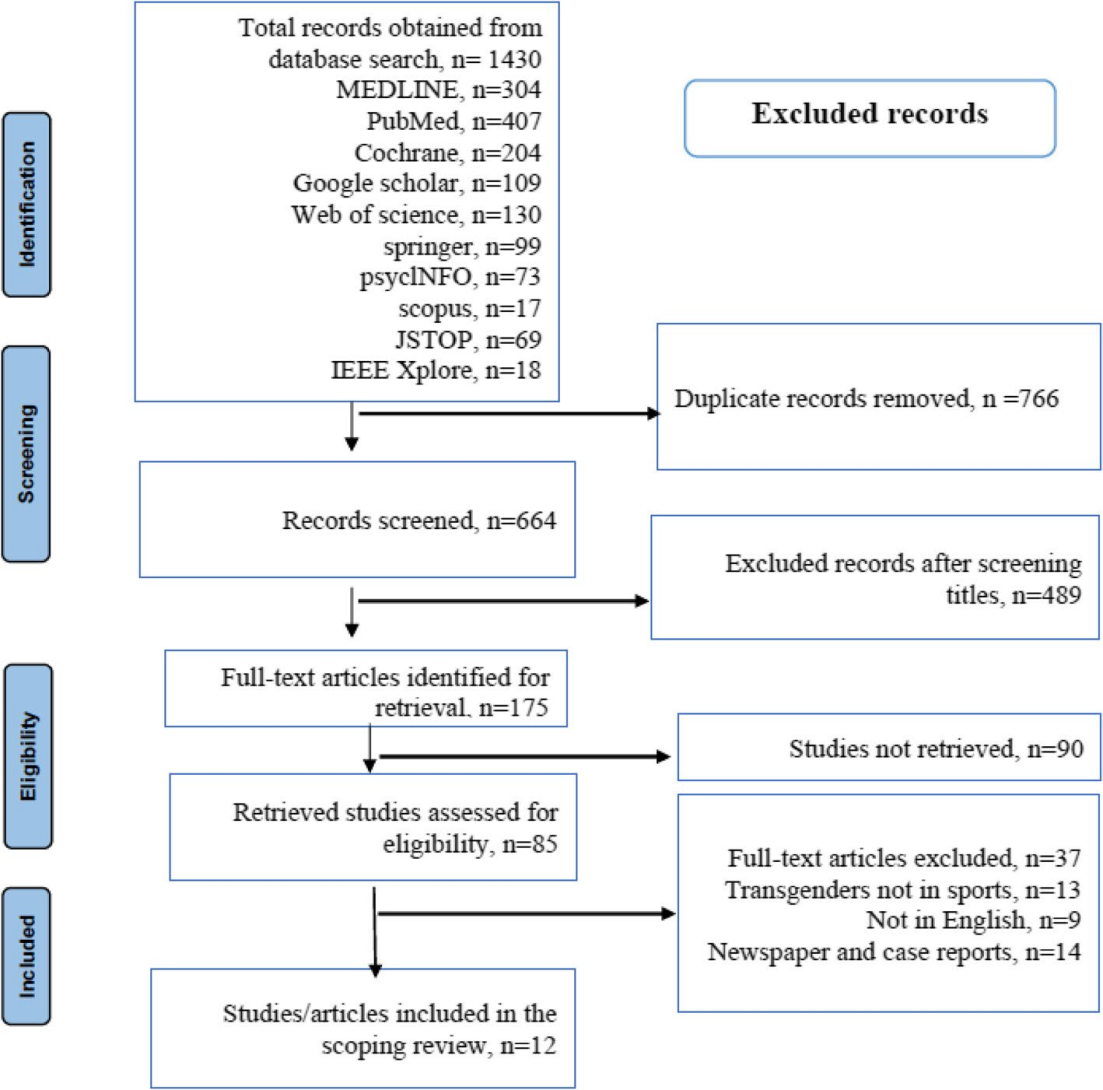
The PRISMA search strategy flow chart of included and excluded studies

A detailed search strategy was employed to analyze the primary articles on inequality and mental health challenges among transgender people in sports by navigating the identified electronic databases. The search was conducted according to the PRISMA guidelines (PROSPERO registered ID: CRD42022329236) (Fig. 1) [34]. After searching the titles of systematic reviews using relevant keywords, 1430 studies were retrieved from the electronic databases. Searches using keywords and terms were effectively performed by modifying them in various electronic databases. A.S.W.C., P.M.K.T., and L.M.L. screened the articles to ensure that only relevant studies were included in this review. Relevant information from the included articles were compiled for evaluation and analysis in this systematic review and meta-analysis. The PRISMA flowchart in Fig. 1 summarizes the search strategy within the explored databases to obtain the 12 studies which were finally included.

### Quality assessment

A quality appraisal of the 12 included studies that met the inclusion criteria was conducted to assess the methodological quality based on the Effective Public Health Practice Project (EPHPP) tool. Two reviewers (P.M.K.T. and L.M.L.) evaluated the accuracy and quality of the included studies. The assessment was based on the six-score domain provided by the EPHPP tool, which has the following study design: data collection methods, dropouts and withdrawals, confounders, blinding, and selection bias. Finally, the study quality was classified as moderate, weak, or strong.

### Data extraction

The included articles were reviewed and analyzed, and essential information was retrieved and compiled. Three reviewers (A.S.W.C., P.M.K.T., and L.M.L.) assessed the relevant data from the included studies and determined the information to be extracted. Discrepancies in the data extraction process were resolved through discussion. The extracted information and data included the study identification (ID) (authors and years of publication), study design, participant characteristics (number of participants, age, citizenship, and gender), study aim, and primary outcomes of the study (participation in sports, inequalities, and mental health outcomes).

### Data analysis

A meta-analysis was conducted on the pooled data using R statistical analysis software (version 4.2.1). A random-effects model was considered and implemented because it was suitable for efficiently considering the sample size of each study with heterogeneity. Given that the sample groups analyzed in this study were obtained from larger populations, paired sample t-tests were used for statistical analysis. The analysis was based on the variation in participants being discriminated against and subjected to mental health problems. Therefore, the inequality and healthcare disparity among transgender athletes was evaluated based on discriminated and welcomed transgender individuals with a 95% confidence interval (CI). Data heterogeneity was determined using I^2^ statistics, in which 25, 50%, and > 70% were considered low, moderate, and significant, respectively. Additionally, a *P*-value < 5% (0.05) represented a statistically significant difference in the meta-analysis.

## Results

### Study characteristics

A total of 1430 studies were identified from diverse electronic databases, and through a systematic screening process, 12 studies met the predetermined inclusion criteria and were selected for this review (Table 1). The majority of these studies employed qualitative research methods, with interviews being the most frequently utilized (*n* = 5), followed by surveys (*n* = 4), cross-sectional surveys (*n* = 2), and qualitative phenomenological studies (*n* = 1).

**Table 1:** The characteristics of included studies

Authors	Study design	Participants	Study objectives/intervention	Associated study outcome
Nye et al. (2019) [35]	Cross-sectional survey	A total of 1077 university and collegiate athlete trainers (ATs), comprising 420 males and 653 females, participated in this study. The highest degree earned by a total of 1074 athletes were as follows: 11.5% had an academic doctorate, 1.4% had a clinic doctorate, 73.6% had a master’s, and 13.6% had a bachelor’s degree.	The main aim of this survey was to explore ATs’ perception of treating LGBT (lesbian, gay, bisexual, and transgender) athlete patients. Using the Qualtrics software, a 19-item questionnaire assessed and with analyzed the approach, the care quality, and perceived behavior of athlete trainers with transgender student-athletes.	A cohort of 78.9% (844) participants revealed that they had a close friend or family member disclosing as transgender or LGBT, and 15.5% (167) identified themselves as LGBT. A majority reported they had not received formal training on the needs of transgender people (44.8%, 482); only a few said they had formal training on the needs of transgender people (9.6%, 103). The mean + S.D.D. (range) of participants reporting an approach change when serving transgender people was 1.15 ± 0.56 for 597 female participants and 1.18 ± 0.58 for male participants; for quality of health care and comfort in providing service, 4.68 ± 0.71 (for females) and 4.60 ± 0.85 (for males).
Calzo et al.(2014) [36]	Survey (Growing Up Today Study)	A total of 16,882 participants were recruited for this study; 939 were females and 7843 were males, and their ages ranged from 12 to 22 years. All participants were from the United States.	The survey explored low self-esteem among transgender athletes, their sports involvement, and team participation.	Sexual minorities reported MVPA (moderate-vigorous physical activities) of *p* < 0.01 (1.21–2.62) and 46–76% were less likely to participate in sports than heterosexuals of the same gender. Athletes’ self-esteem contributed to approximately 46–100% of sexual identity MVPA differences.
Elling-Machartzki (2017) [37]	Interview	Twelve transgender individuals from the Netherlands participated in this study. Six were transgender men and six transgender women. Their age varied from 27 to 51 years	Challenging physical activities and sports in the lives of transgender individuals concerning the centrality of their bodies.	The 12 transgender women and men reported being forced to fit in or be excluded from some competitive sports, especially during the transition process. They expressed sports as disciplining, shaming, and dangerous for transgender individuals. However, a few participants were happy and felt empowered in supportive social environments.
McGannon et al. (2019) [38]	Conversational interview	Ten boxers from the women’s Canadian national team were recruited, aged 26 to 31 years, with a mean of 28.3 years. Five identified themselves as transgender, bisexual, or lesbian, and five as heterosexual. All participants had competed a world championship, four being world champions with medals, six having major games medals, and three having competed the Olympics on the Canadian team.	The study examined the psychological impact among transgender boxers in sports by employing a face-to-face video interview over Skype. Participants shared their performance, well-being, competition experiences, training connections, and the impacts of their identities.	50% of participants who responded were transgender boxers on the Canadian women’s team. The majority showed significant social-psychological effects.
Travers (2006) [39]	Open-ended interview	Twenty women from the NAGAAA (North American Gay Amateur Athletic Alliance) participated in this study.	Exploring softball leagues’ responses to challenges encountered by transgender people, and transgender-inclusive policies.	Most leagues have not solved the transgender issues in their legal policies in explaining the eligibility for participation. One transgender participant revealed that many women are not subjected to these policies and referred to it as not safe for transgender people. Another respondent indicated that men who transitioned into women still had more strength and could harm the rest of the players, referring to them as physically superior.
Travers and Deri (2011) [40]	Interview	A total of 12 individuals who identified as transgender, including eight transgender men, three transgender women, and one lesbian, participated in this study.	An interview was conducted to measure the inclusivity of transgender people in sports by employing a climate metaphor system developed by Sandler and Hall.	The eight transgender men reported that they were warmly accepted and faced no discrimination.
Hargie et al. (2017) [11]	Interview	Ten transgender individuals were recruited for an interview in this article. Six were females, and four were males. Their ages ranged from 25 to 62 years. All participants were from Northern Ireland.	Through a focus group, the identity Trust, a support group in Belfast conducted interviews with transgender people to explore their interests in sports, inclusionary and exclusionary experiences, their emotions in sports, and the impact of being transgender in sports. Interviews lasted between 30 minutes and one hour and were conducted at Ulster University.	Transgender people felt discomfort in changing rooms. They were told they were neither one gender nor the other and felt excluded. They reported negative experiences at school. They were humiliated in sports by being told to prove their gender identity for two or more years before joining sports teams.
Mereish and Poteat (2015) [15]	Survey	A total of 13,933 student participants from twenty-two high schools in Dane County were enrolled in this survey.	To examine sexual identity disparities in physical activities in sports among transgender individuals.	Among the LGBT participants, 8% were physically active; 57% of heterosexual and 35% of LGBT participants were involved in sports. (AOR = 1.28; 95% Cl = 0.46, 0.83) Heterosexual males were more likely than LGBT males to be physically active or involved in sports (AOR = 0.26; 95% CI = 0.20, 0.32).
Walen et al. (2020) [41]	Cross-sectional survey	A total of 5303 participants were eligible for the survey. Eight hundred ninety-four participants started the survey (response rate was 16.2%), and 667 identified as transgender in their own words (completion rate was 74.6%). Participants were 33 ± 10 years of age, and their average experience was 11 ± 10 years.	The measure of athlete’s trainers perceived defining of transgender people, comfortability working with transgender people, and competitive advantages perceptions. A multi-process 43-item questionnaire was employed to explore the objectives.	321 (48.1%) of the recruited individuals reported receiving transgender treatment, although 240 (36%) said they were in the process of screening drugs in consultation with an endocrinologist. 45.6% (304) of participants reported they were competent in using suitable technology related to transgender people. 321 (48.1%) disagreed that they were competent to counsel transgender people regarding replacement effects associated with hormones or mental health issues (*n* = 269, 40.3%). 35.1% (243) reported never having received education on caring for transgender patients, 278 (41.2%) believed that transgender females had more competitive advantages, while 44 (6.6%) thought male transgender athletes had more competitive advantages.
Munson and Ensign (2021) [42]	Qualitative phenomenological study (survey)	A total of nine individuals were recruited for this study. Six were recruited via a quantitative survey and three through word of mouth. Their age ranged from 18 to 35 years, with a mean age of 23.56 ± 5.32 years, and they were currently athletes or had been so in the last five years. Three were transgender women, four identified as gender-queer or nonbinary, and one identified as nonbinary and transgender male. Two were athletes in high school, four in collegiate, one semiprofessional, and two in league sports. Three were transgender male.	To examine challenges or experiences that ATs face and barriers transgender athletes face in sports. Interviews were conducted to find the primary objectives.	Most transgender people believe that ATs must be trained on different subjects concerning transgender individuals. Five reported that their ATs had insufficient knowledge of transgender people’s needs. One transgender individual said the ATs seemed ignorant when they did not know how to interact with him as a transgender athlete.
Herrick and Duncan (2018) [43]	Survey	A cumulative 42 individuals participated. All participants were 18 years and above and could speak and read English. The mean age was 28 years, and they were from Canadian cities. Participants identified as: twelve queer, eleven gay, nine lesbian, six bisexual, two polysexual, one asexual, and one not specified. The gender identities were: cis-men 16; cis-women 15; five non-binary; two gender-fluid; two transgender men; one transgender woman; and one agender individual. The majority were white (31); the rest were four Asians, four Arab, two Hispanics, and one Black.	The survey employed eight focus groups and semi-structured interview guidelines to examine challenges such as exclusion and discrimination among transgender people in sports and to explore their experiences and participation in physical activities as transgender athletes. All the focus groups had between three to eight individuals and were held between 55 minutes to 95 minutes.	Cis-women individuals felt they did not belong there and were not warmly welcomed in physical activities. Most participants reported negative experiences in changing rooms in the context of physical activities, and few had positive experiences. LGBT individuals said they felt shameful when entering locker rooms, were transgressing heteronormative interests and believed it was wrong. One transgender person, a 32-year-old, reported feeling fear and being discriminated against at the gym because they were misgendered.
Lucas-Carr and Krane (2012) [44]	Interview	Three transgender individuals participated in this study; all participated in high school sports, and two continued to participate in the collegiate club. In contrast, the others participated in non-university club sports. One participant, identified as Ryan, was 28 years of age; another, Harvey, was 29 years; and Jake was 23 years of age. All were white and lived in the United States.	The three transgender people were interviewed to explore athlete transgender experiences in sports using a life history methodology system. The themes examined were sex segregation and sport, the safety space in sports participation, and the inclusion of transgender people in sports.	One transgender individual, Harvey, revealed sex segregation as a frustration in sports participation. Ryan reported that few options were available in sports because of his identity. Jake expressed how being different is often seen as threatening. The three individuals were positioned to secure space in sport as they pushed the transgender boundaries. Harvey was frustrated when he joined the women’s rugby team because his friends frequently pointed out that he was on a girls’ team. All participants felt they were not well-accepted in sports, as Harvey constantly said sex segregation should be abolished in sports.

Note: ATS athlete trainers, *MVPA* moderate-vigorous physical activities, *NAGAAA* North American Gay Amateur Athletic Alliance

### Study quality assessment

EPHPP tool domain rating analysis revealed that two studies were of strong quality [34, 35], four of moderate quality [18, 21, 33, 37], and six of weak quality [22, 30–32, 36]. The domain ratings of the EPHPP tool for various studies are illustrated in Table 2.

**Table 2:** EPHPP tool domain ratings

Study	Global rating	Selection bias	Study design	Confounders	Blinding	Data collection methods	Withdrawals and dropouts
	Strong*: 1 ≤ weak rating* Weak*: > 1 weak rating*	Strong*: participants more likely to represent the target population with > 80% participation.* Weak*: less likely to represent the population with < 60% participation.*	Strong: *randomized/clinical controlled trial* Weak*: any design apart from randomized, clinical, case-control, cohort anality, and cohort interrupted time series.*	Strong*: controlled for at least 80% of relevant confounders* Weak*: controlled for less than 60% of relevant confounders.*	Strong*: outcome assessors unaware of participants’ intervention status AND participants unaware of the research question.* Weak*: aware of both.*	Strong*: valid and reliable data collection methods* Weak*: data collection methods both unreliable and invalid.*	Strong*: greater than 80% follow-up rate.* Weak*: follow-up rate of less than 60%.*
Agnes Elling-Machartzki (2017) [22]	Weak	Strong	Weak	Moderate	Weak	Weak	Strong
Calzo et al.(2014) [21]	Moderate	Moderate	Weak	Moderate	Strong	Moderate	Moderate
Hargie et al. (2017) [32]	Weak	Moderate	Weak	Weak	Moderate	Strong	Strong
Herrick and Duncan (2018) [36]	Weak	Weak	Weak	Moderate	Weak	Moderate	Strong
Lucas-Carr and Krane (2012) [37]	Moderate	Weak	Weak	Moderate	Moderate	Strong	Strong
McGannon et al. (2019) [30]	Weak	Weak	Weak	Weak	Strong	Strong	Weak
Mereish and Poteat (2015) [4]	Weak	Strong	Weak	Moderate	Weak	Moderate	Weak
Munson and Ensign (2021) [34]	Strong	Strong	Weak	Strong	Moderate	Strong	Strong
Nye et al. (2019) [18]	Moderate	Strong	Weak	Strong	Moderate	Moderate	Strong
Travers (2006) [31]	Weak	Weak	Weak	Moderate	Strong	Moderate	Strong
Travers and Deri (2011) [35]	Strong	Strong	Weak	Moderate	Strong	Strong	Strong
Walen et al. (2020) [33]	Moderate	Strong	Weak	Strong	Moderate	Moderate	Moderate

### Meta-analysis

**Fig. 2:**
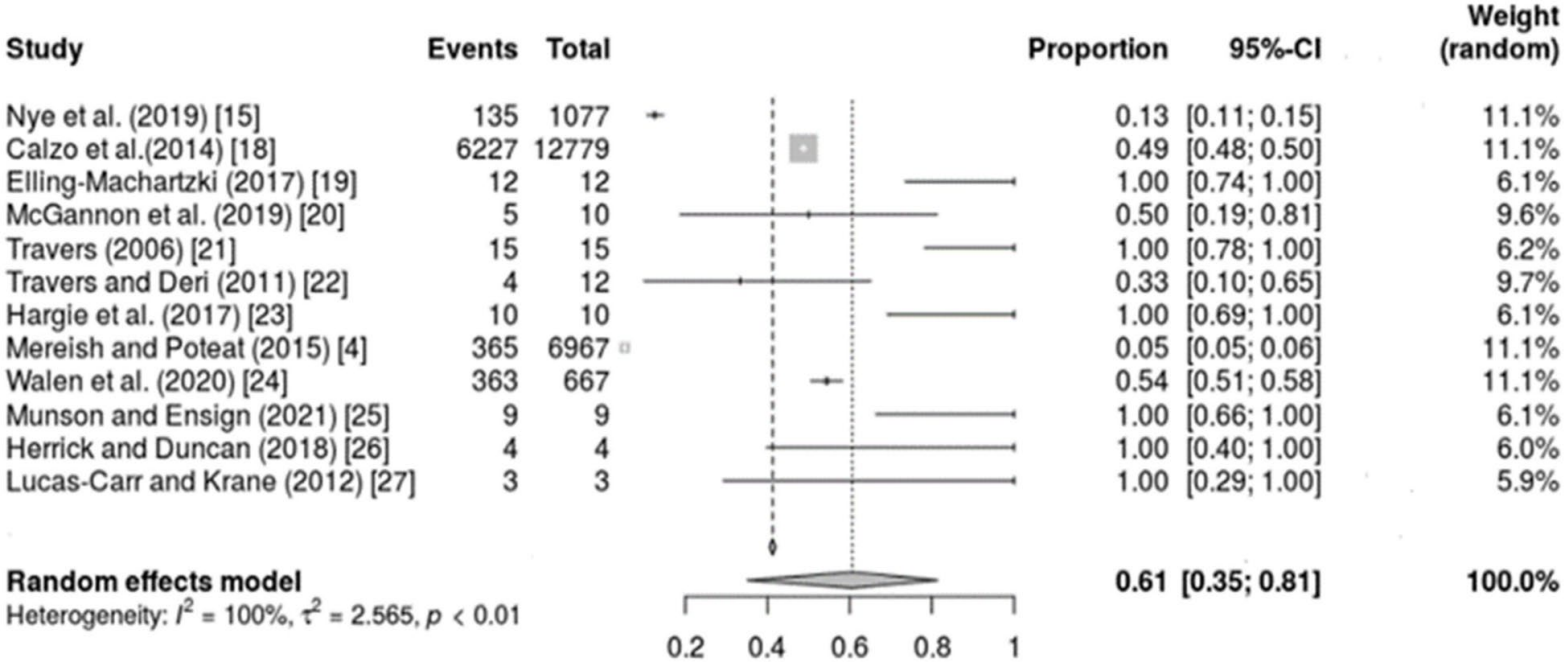
Forest plot indicating the discriminated transgender athletes

A group meta-analysis of the results from the 12 studies on the discrimination of transgender athletes indicated substantial heterogeneity (I^2^ = 100%;) (Fig. 2). The high heterogeneity was due to the significant variation in outcomes among the studies (variation in participants subjected to discrimination).

**Fig. 3:**
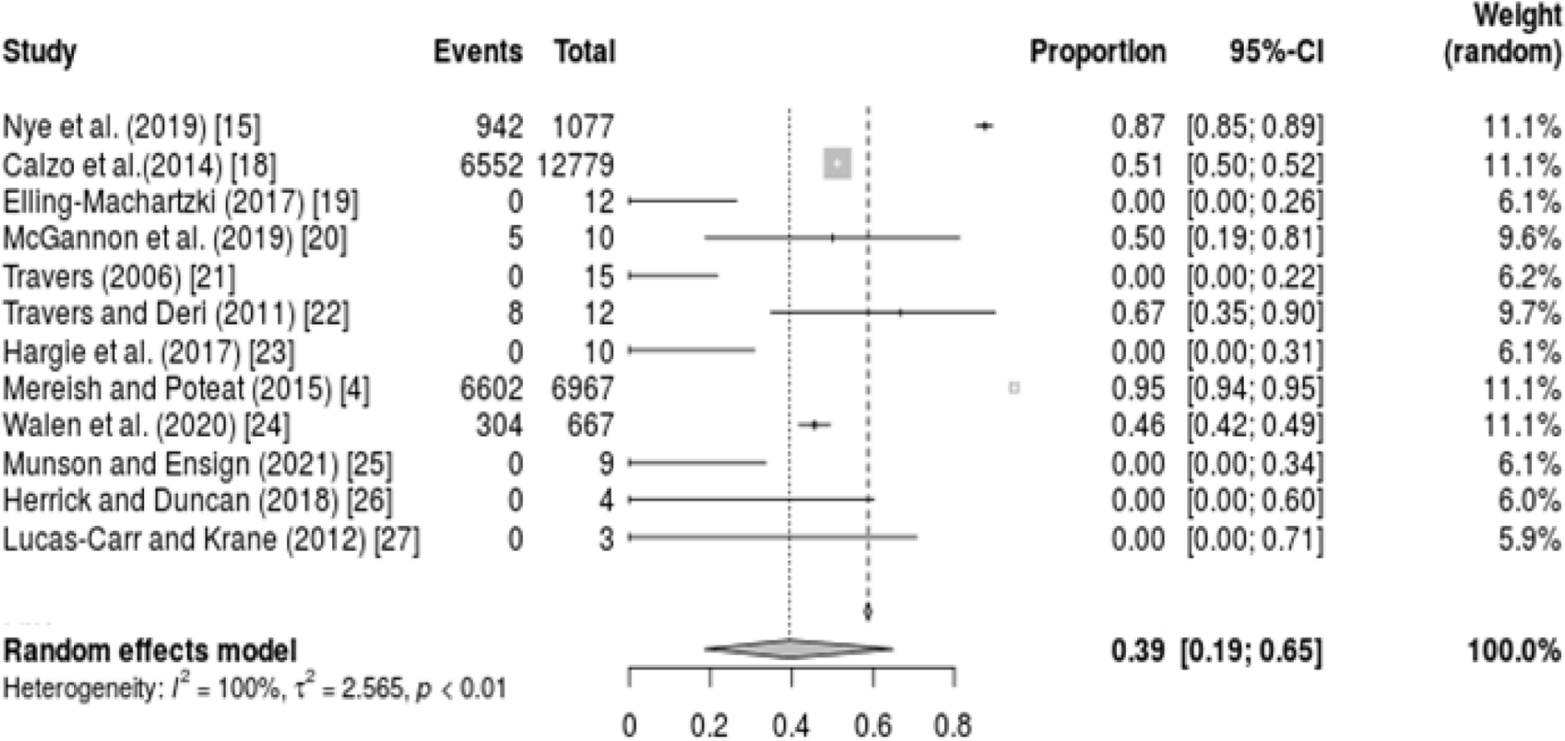
Forest plot showing transgender athletes welcomed into the sporting activities

Similarly, a group meta-analysis of transgender athletes who were welcomed and appreciated in sporting activities indicated high heterogeneity (I^2^ = 100%) (Fig. 3). Such significant heterogeneity was due to the wide differences in the outcomes among the included studies.

**Fig. 4:**
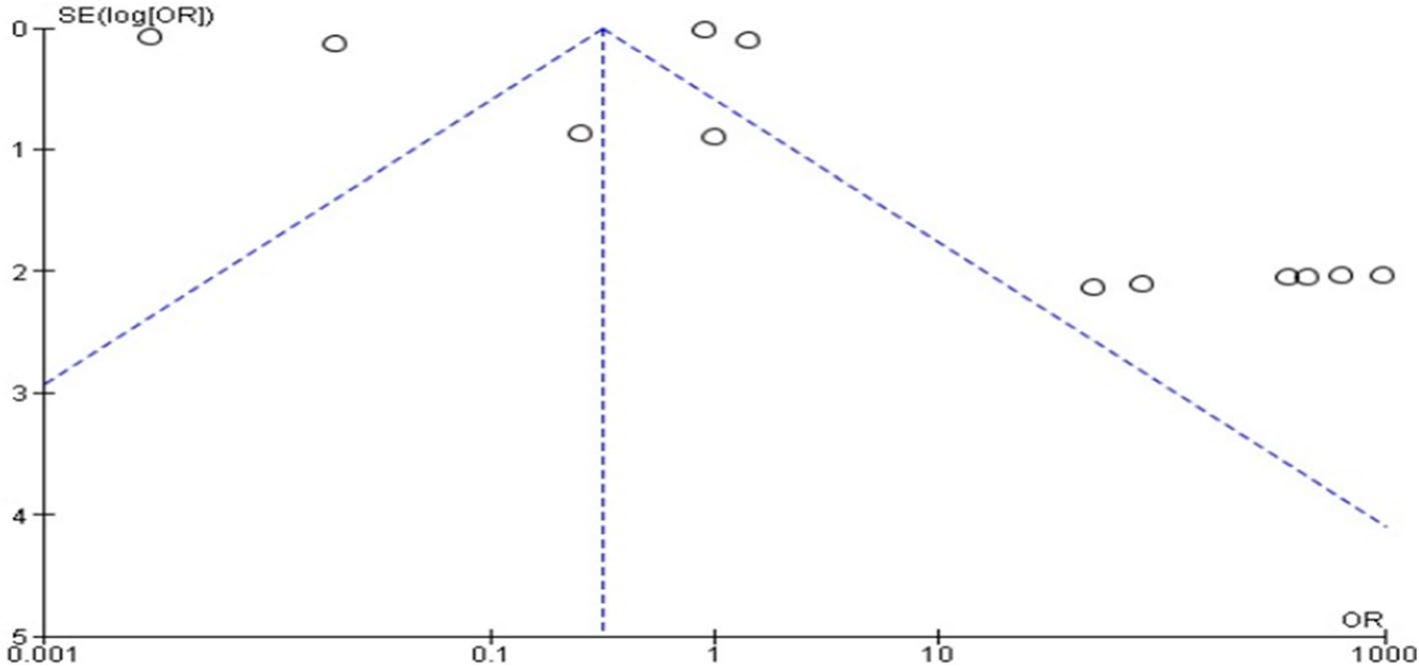
Funnel plot

The asymmetry in the generated funnel plot (Fig. 4) also showed variations in the study outcomes due to differences in the quality of the included studies.

## Discussion

Transgender individuals reported negative experiences in sports, particularly in competitive sports, and felt discomfort in changing rooms. Many participants believed that leagues have not solved transgender issues in their legal policies, and that ATs must be trained on different subjects concerning transgender individuals. Additionally, a significant proportion of participants reported not feeling competent in counseling transgender people regarding replacement effects associated with hormones or mental health issues.

A meta-analysis of the 12 included studies indicated that, from the cohort of 21,565 participants in the studies included in this review, most of which comprised transgender athletes, 7152 (33%) were subjected to discrimination in sports participation as well as in healthcare. The inequality and healthcare disparities that transgender athletes faced account for 0.61 (95% CI: 0.35, 0.81), as shown in Fig. 2. On the other hand, the transgender athletes who felt welcomed and embraced in their respective teams accounted for 0.39 (95% CI: 0.19, 0.65) as shown in Fig. 3. Statistically comparing these two aspects, there is a relative difference between how transgender athletes are treated both in sports and when seeking medical attention, even by athlete trainers.

Most of the studies included in this article focused on exploring the mental health and inequality among transgender individuals in sports. Two studies evaluated the perception of ATs regarding transgender athletes by employing a quality care approach associated with comfort when treating them [11, 42, 45]. The results showed that most ATs had a significantly positive perception of their transgender patients [32, 46]. However, differences in gender, relationships, and religion were observed [45]. Thus, some ATs had standard norms for the general population [35]. Some ATs showed judgment and discrimination toward transgender people, while the majority demonstrated insufficient skills to care for and attend to transgender athletes and patients [31, 42, 47, 48]. Participants advocated for training among Ats regarding ‘the needs of transgender people and for creating awareness to solve inequalities in sports [38, 39]. Moreover, the lack of experience was motivated by the uncomfortable, unhelpful, and hostile treatment of transgender individuals in sports, and they felt excluded from participation [11, 49]. Studies examining the physical involvement among transgender people and those with other sexual orientations have indicated that transgender people are less likely to engage in physical activities and sports teams than their heterosexual counterparts [15, 37]. One study reported that transgender people were discriminated against at an early stage according to moderate-vigorous physical activity (MVPA) findings; this was found to be a common challenge among transgender people in sports participation [37, 42, 50].

Moreover, three studies highlighted evidence that transgender athletes experienced mental health and inclusive challenges when participating in games in schools and other clubs [38, 41, 44]. In particular, participants’ self-esteem was one of the key mental health issues among different sexual minority identities, with the majority showing less positive perceptions of their participation in athletics [15]. Therefore, some transgender MVPAs change as they grow and do not participate in sports teams [36]. Sexual minorities are likely to be more active in physical activities. They participate in sports at an early age, but because of frustrations from ATs and other sexual identities within their teams, they start showing less interest in participation [32, 42, 51]. Most of these disparities cause significant issues because physical activities, such as involvement in sports, can reduce health risks among transgender people. Thus, most are likely to be at a high risk of depression, suicide, drug abuse, and mental health disorders [15, 52]. It was reported that approximately 50% of transgender men and 30% of transgender women attempted suicide at one time, and therefore they needed mental health care. If trainers are provided with care skills, transgender people have greater access to mental health services access [42, 53].

Effective training and emphasis should be encouraged on the roles of AT management in the psychological health among transgender individuals so that they can interact with the system more freely and experience less or no discrimination [42, 43]. A participant in one of the studies, named Ryan, expressed how transgender people have limited options in sports; their experiences show a deep belief concerning different gender orientations and sports [43, 54]. Despite many transgender athletes reporting negative experiences with sports participation, some experienced a positive interaction [13, 37].

## Recommendations

### Policy reforms to ensure inclusion and fairness

To address the issues and discrimination faced by transgender individuals in sports, it is vital to implement a comprehensive approach that encompasses policy reforms, training, awareness, and mental health support. Policy and legal reforms should be given top priority to safeguard the rights and inclusion of transgender individuals in sports. Sporting leagues and organizations must take the lead in formulating and enforcing policies that cater to the specific needs of transgender athletes [55]. These policies should guarantee equal opportunities for participation, access to appropriate changing facilities, and protection against discrimination [56]. By establishing clear guidelines, we can create a more inclusive environment that respects and supports transgender athletes.

In particular, the most common question faced by individuals within the sports domain will likely revolve around determining when it is safe and fair to permit a transgender person to compete in sports based on their experienced gender. Presently, this remains a complex issue to address due to the absence of direct and consistent physiological performance-related data for transgender individuals [57]. This lack of data hampers the ability to reach a consensus on whether transgender people, especially transgender females, possess an athletic advantage. Given these circumstances, it may be prudent to suggest that until there is direct and consistent scientific evidence supporting the notion of transgender competitors having an advantage, transgender individuals should be allowed to compete in accordance with their gender identity without restrictions, such as the absence of requirements for cross-sex hormones or gender-confirming surgery [58].

Moreover, the athletic advantage perceived in transgender female individuals, based on indirect and inconclusive evidence, may not be more significant than the widely accepted physiological advantages, such as large hands, and financial advantages, such as access to extensive training opportunities, enjoyed by some cisgender individuals in competitive sports [58]. Therefore, for sports organizations seeking to exclude transgender individuals from competing in their experienced gender category, they would need to demonstrate that the sport is significantly influenced by gender, and that such exclusion is necessary to ensure fair and safe competition. Currently, this would be challenging, given the lack of evidence suggesting that androgenic hormone levels consistently confer a competitive advantage.

### Training programs for health professionals in sports

A second essential component is the implementation of specialized training programs to educate athletic trainers (ATs) on transgender issues. ATs should undergo comprehensive training that covers various aspects, including transgender healthcare, the effects of hormone replacement, mental health support, and counseling techniques [59, 60]. This training equips ATs with the necessary knowledge and skills to understand and address the unique needs of transgender athletes, ensuring they receive appropriate care and support. Healthcare professionals working in the field of sports must also familiarize themselves with the specific challenges and concerns that transgender individuals may encounter when participating in sports [61].

### Awareness and education initiatives

Furthermore, raising awareness and promoting education about the challenges faced by transgender individuals in sports is essential. Organizing workshops, seminars, and educational campaigns is critical to enhancing awareness among ATs and the general population [62, 63]. These initiatives should focus on promoting inclusivity, respect, and understanding. By creating a more inclusive environment, transgender athletes will feel welcomed and embraced in their respective teams, leading to a more positive sporting experience.

### Encouraging physical activity

Encouraging physical activity among transgender individuals is also of paramount importance [64, 65]. Efforts should be made to create safe and supportive environments that address discrimination and biases. Promoting the benefits of physical activity for overall health and well-being can help increase participation among transgender individuals. By fostering a positive and inclusive environment, we can encourage transgender athletes to engage in physical activities and join sports teams [55]. This holistic approach will pave the way for a more inclusive, equitable, and positive sporting environment for all, regardless of gender identity.

## Conclusion

The study found that while most ATs had a positive perception of their transgender patients, discrimination and prejudice in athletes’ training were highlighted. The participants demonstrated a sense of responsibility and professionalism, but the outcomes were mixed, with some ATs treating transgender patients professionally and others offering unprofessional assistance in navigating their sexuality in relation to sports participation. Health interventions should prioritize promoting physical activity and sports participation among sexual minorities such as transgender people, creating spaces for transgender athletes to participate in competitive sports environments, and developing federal policies to abolish bullying of sexual minorities in schools and clubs. The perception of gender minorities in sports should be reviewed to include different gender orientations, and gender minorities should feel accepted and comfortable interacting with other participants in their chosen sports.

## Data Availability

All data generated or analysed during this study are included in this published article.
